# African Pediatric Nephrology Guidebook: Coordinator/Scientific Editor Amal Bourquia: IPNA 2015, 1st edn, ISBN 978-9954-9062-5-5

**DOI:** 10.1007/s00467-018-3897-3

**Published:** 2018-03-06

**Authors:** Mignon Irene McCulloch

**Affiliations:** African Paediatric Nephrology Association, Paediatric Nephrology and Paediatric ICU, Red Cross Children’s Hospital, Klipfontein Road, Cape Town, Western Cape 7700 South Africa

This unique book, published in 2015, addresses paediatric nephrology issues in Africa and is available with both English and French text. What makes it different to any other existing handbook is that it addresses specific renal pathological conditions that may not be seen in other well-resourced parts of the world with practical advice, yet each chapter is well referenced. It also has input from a diverse group of authors, not only based in various regions in Africa, but also some invited authors from further afield.

The main themes that form the basis of the book include:

1. Nephritic/nephrotic issues, which are probably the most common renal conditions seen by nephrologists in Africa. The book addresses issues of diagnosis including renal biopsies where they are available. A wide variety of therapies are discussed for both steroid-sensitive and steroid-resistant nephrotic syndromes, including the most recent therapies such as rituximab, if a centre has funding and access to this. There is also a chapter on the genetics of nephrotic syndrome.
2. Other conditions that are also prevalent in Africa include lupus, sickle cell, and HIV, and local experience with these in terms of diagnosis and treatment is explored.
3. Tubulopathies are also discussed with specific emphasis on nephrolithiasis, which is more common in northern parts of Africa.
4. The text reviews acute kidney disease (AKI), including more unusual conditions for well-resourced areas, but that may be prevalent in certain parts of Africa, including infections such as malaria, typhoid, and snake bites in addition to a chapter on haemolytic uraemic syndrome (HUS). HUS is discussed as pertains to the D+ HUS variety that is commonly seen, but also the D−, more atypical form, and the investigations required around this type including complement and genetics.
5. There is a brief section on dialysis, which is relevant in view of the diversity of the available facilities in Africa. All forms of dialysis are touched upon, considering that some isolated centres in the have no access to dialysis, whereas others compare favourably with centres in well-resourced areas elsewhere in the world.
6. The section on urology has five chapters, which start with antenatal diagnosis issues in an African setting, voiding disorders and urinary tract infections, in addition to posterior urethral valves.

Dr Bourquia has done well in putting this book together, gathering expert opinion from across the African continent while providing practical advice in a local setting. She ends the book by relating all the renal issues to the African setting, particularly to the French-speaking areas.

The book concludes with a good glossary of useful norms in paediatric nephrology that will be very useful for the junior doctor or nurse working in an African setting.

Date of review: 19 September 2017

